# Effect of an Education Program on Improving Help-Seeking among Parents of Junior and Senior High School Students in Japan

**DOI:** 10.5539/gjhs.v4n1p33

**Published:** 2012-01-01

**Authors:** Hatsumi Yoshii, Yuichiro Watanabe, Hideaki Kitamura, Zhang Nan, Kouhei Akazawa

**Affiliations:** School of Health Sciences, Faculty of Medicine Niigata University, Niigata, Japan Tel: 81-252-272-264 E-mail: yoshii@clg.niigata-u.ac.jp; Department of Psychiatry, Niigata University Graduate School of Medical and Dental Sciences Health Administration Center, Headquarters for Health Administration Niigata University, Niigata, Japan Tel: 81-252-272-213 E-mail: yuichiro@med.niigata-u.ac.jp; Department of Psychiatry Niigata University Graduate School of Medical and Dental Sciences Niigata, Japan Tel: 81-252-272-213 E-mail: kitamura@med.niigata-u.ac.jp; Department of Medical Informatics and Statistics Niigata University Graduate School of Medicine, Niigata, Japan Tel: 81-252-272-471 E-mail: zhangnan@med.niigata-u.ac.jp; Department of Medical Informatics and Statistics Niigata University Graduate School of Medicine, Niigata, Japan Tel: 81-252-272-471 E-mail: akazawa@med.niigata-u.ac.jp

**Keywords:** Help-seeking, Parents, Education program, Schizophrenia

## Abstract

Early intervention in schizophrenia is important for patient prognosis and quality of life. At the time of the first episode, quality of life is influenced by identification of symptoms and by medical help-seeking behavior. In this prospective cohort study, we investigated help-seeking among 2690 parents of junior and senior high school students before and after the parents viewed a newly developed web-based education program aimed at improving knowledge of schizophrenia. Our web-based education program aimed to improve understanding of schizophrenia, including promotion of help-seeking. Many parents (33.1%-50.0%) consulted a physician in a department of psychosomatic medicine when their child experienced symptoms. Characteristics that predicted a decision not to seek psychiatric medical help were having child with all symptoms, younger parent age, and lower family income (p<0.05). After the education program, the rate of parents who sought medical help within 1 week was significantly higher for all symptom categories except sleeplessness (p=0.001). These findings suggest that the present web-based education program was useful in promoting medical help-seeking behavior among parents of junior and senior high school students in Japan.

## 1. Introduction

Early intervention in schizophrenia is important for patient prognosis and quality of life (QOL). Another reason for rapidly beginning treatment is that longer duration of untreated psychosis (DUP) is associated with lower long-term treatment effectiveness (Van *et al.*, 2005). Thus, early intervention might improve response to antipsychotic treatment and long-term outcome (Perkins *et al.*, 2005). Loebel *et al*. (1992) reported that duration of symptoms before treatment was significantly associated with time to remission and level of remission, i.e., longer duration predicted longer time to remission and lesser extent of remission. Longer DUP is associated with mental anguish, declines in QOL and social functioning, and poor clinical outcomes (Bechard-Evans *et al.*, 2007). The average DUP was reported to be between 1 and 2 years (Larsen *et al.*, 1998). Reducing DUP is an important challenge for mental health professionals, as it influences patient prognosis (Chong *et al.*, 2004). In a review by Kessler *et al*. (2005), the authors noted that one-half of cases of mental illness begin by age 14 years and that three-fourths begin by age 24 years. In addition, they found evidence of delays in help-seeking among young people with emerging psychosis (Lincoln *et al.*, 1995; Bechard-Evans *et al.*, 2007). Bechard-Evans *et al*. (2007) showed that adolescents show poor social and academic adjustment and are socially withdrawn. Furthermore, their changes in behavior are more likely to go undetected when psychosis begins. Therefore, they are less likely to be brought to a mental health professional for help. These findings show that rapid detection of the initial onset of psychosis is important in young people because it would permit treatment to start earlier. However, young patients who are severely mentally ill have few mental health consultations (Nishida *et al.*, 2008), and young people who need treatment frequently do not seek help (Boydell *et al.*, 2006). Therefore, psychiatric outcomes among young patients might depend on whether their parents can understand the symptoms of mental illness and seek appropriate medical care at an early stage (Helgason, 1990; Perkins *et al.*, 2005).

In recent years, there have been a number of studies of help-seeking (Platz *et al.*, 2006; Unal *et al.*, 2007; Compton *et al.*, 2008; O’Callaghan *et al.*, 2010). However, there has been no such study among the parents of junior and senior high school students. In this prospective cohort study, we (1) assessed help-seeking among parents when junior and senior high school students have schizophrenia symptoms or prodromal and nonspecific symptoms of schizophrenia, (2) identified factors associated with failure to seek medical help, and (3) investigated the effectiveness of a newly developed web-based education program that aimed to improve understanding of schizophrenia, including the promotion of help-seeking.

## 2. Methods

### 2.1 Participants

The participants were 2690 parents of junior and senior high school students. They were extracted from candidates in a large database administered by a private Japanese company that specializes in questionnaire research. Gender and region were used as variables for stratified random sampling. Consent was obtained from all participants by the same company that administered the database. All participants completed a questionnaire on an internet website administered by the survey company. The details have been previously described (Yoshii *et al.*, 2011). This study was approved by the Ethics Committee of the Niigata University School of Medicine.

### 2.2 Questionnaire

The questionnaire used in the present study consisted of 3 sections. Section 1 collected demographic information on respondents. Section 2 asked about consultations their child had for a symptom of schizophrenia, a prodromal symptom, and nonspecific symptoms of schizophrenia. The participants were then asked to indicate all types of consultations they had sought from among 15 choices (e.g., family circle, homeroom teacher, psychiatric clinic, health center) for a child with sleeplessness (nonspecific symptom of schizophrenia), social withdrawal (prodromal symptom), strange behavior (symptom of schizophrenia), or all 3 symptoms. In section 3, the participants were requested to select from 5 items regarding the timing of the consultation with regard to onset of symptoms (within 1 week, about 1 month later, about half a year later, more than 1 year later, treatment not needed) when their child had the above symptoms (the first questionnaire). All participants then viewed the education program. One week later, the questionnaire was answered again (the second questionnaire), and the effectiveness of the education program was evaluated among the participants.

### 2.3 Web-based education program

After completing the first questionnaire survey, all respondents were invited to view a web-based education program that aimed to improve understanding of schizophrenia, including promotion of help-seeking. This program was developed by the authors (Yoshii *et al.*, 2011). The content included help-seeking, i.e. how to prevent progression and exacerbation of the disorder, signs of progression, and consultation alternatives. The education program comprised 12 slides with narration and required 13 minutes to complete. The education program was delivered via the same internet website that was used for the questionnaire survey.

### 2.4 Statistical analysis

All analyses were performed using the Statistical Package for the Social Sciences (SPSS) version 16.0. McNemar’s test was used to compare paired data, i.e., the results of the first and second questionnaires for each respondent. The chi-square test was used to compare both the characteristics of those seeking non-medical help and several demographic characteristics. Differences in rates between groups were assessed with the Bonferroni multiple comparison procedure. All statistical tests were 2-tailed and statistical significance was defined as a P value less than 0.05.

## 3. Results

### 3.1 Characteristics of participants

The participants where 2690 parents of junior and senior high school students in Japan, 2465 of whom finished both questionnaires. Mean age ±SD was 45.9 ±4.7 years. A total of 2552 (94.9%) respondents reported being married. Most (51.0%) respondents were employed full-time. The detailed characteristics of the respondents have been previously described (Yoshii *et al.*, 2011).

### 3.2 Medical help-seeking behavior among parents of junior and senior high school students

Table 1 shows the rate of help-seeking behavior, by type of consultation, reported on the questionnaires given before and after the education program. The most frequent (33.1%-50.0%) type of consultation selected by participants was one at a department of psychosomatic medicine. Only 6.5% to 17.3% of participants with children who had the 4 investigated symptoms chose to have a consultation in a mental hospital. The rate of parents seeking help was similar among those with children who showed all 3 symptoms and those with children who showed strange behavior. Thus, strange behavior was the conclusive factor in seeking medical help.

**Table 1:** Rate (%) of help-seeking among parents of junior and senior high school students

	Sleeplessness (A)			Strange behavior (B)			Social withdrawal (C)			A, B, and C		
Type of consultation	First	Second	p*	First	Second	p*	First	Second	p*	First	Second	p*
**Medical**												
Mental hospital	6.5	6.8	0.822	14.2	14.2	0.717	8.7	8.9	1.000	17.3	17.8	0.941
Psychiatric clinic	13	13.8	0.592	24.1	26.8	0.051	15.9	18.6	0.017	27.4	31.4	0.003
Department of psychosomatic medicine	33.1	29.9	0.024	45.2	43.3	0.319	35.1	36.0	0.532	50.0	51.0	0.313
Department of internal medicine	21.6	17.3	0.001	5.9	5.7	0.952	2.7	2.8	0.931	5.5	5.1	0.609
**School**												
Homeroom teacher	13.9	19.8	0.001	23.6	31.2	0.001	41.4	45.4	0.001	29.0	35.0	0.001
School nurse	8.5	13.4	0.001	10.8	16.4	0.001	12.5	19.3	0.001	11.5	18.7	0.001
School counselor	10.5	13.8	0.001	16.5	21.7	0.001	27.5	30.3	0.011	20.9	26.1	0.001
**Community**												
Health center	1.9	2.7	0.055	2.8	4.8	0.001	2.1	4.1	0.001	3.1	5.6	0.001
Mental health center	2.7	4.3	0.004	6.2	9.7	0.001	5.2	8.9	0.001	7.8	12.1	0.001
**Other**												
Family circle	53.6	61.2	0.001	49.9	56.8	0.001	51.8	57.5	0.001	48.7	54.9	0.001
Neighbor	1.6	1.9	0.445	1.0	0.6	0.511	1.0	0.9	1.000	0.8	0.7	0.868
Classmate’s parents	6.6	7.6	0.058	4.6	4.6	0.632	6.7	6.5	0.813	4.5	4.2	0.944
Telephone consultation	5.5	4.7	0.323	8.3	7.0	0.227	8.9	7.5	0.232	9.0	7.8	0.314
Internet consultation	10.8	8.5	0.022	13.3	11.0	0.034	14.1	11.1	0.004	13.8	11.6	0.057
Needless treatment	6.7	6.8	0.824	4.4	4.7	0.737	4.8	4.9	1.000	4.2	4.5	0.730
**Timing of medical help-seeking**												
Within one week	31.9	34	0.150	40.3	46.0	0.001	24.4	30.8	0.001	44.9	51.4	0.001
About 1 month later	53.3	54.3	0.340	46.8	44.9	0.242	55.5	54.8	0.688	43.7	39.9	0.012
About half a year later	7.5	5.8	0.011	8.3	5.5	0.001	13.2	9.4	0.001	7.1	4.8	0.001
More than 1 year later	1.8	1.1	0.042	1.4	0.9	0.149	2.2	1.7	0.358	1.5	1.4	1.000
Non-medical help sought	5.5	4.9	0.228	3.2	2.6	0.132	4.7	3.2	0.005	2.9	2.5	0.294

* McNemar’s test

The same questionnaire was administered to the participants 1 week after they had viewed the education program on the website. The rates of those who reported seeking a consultation at a psychiatric clinic for children with social withdrawal or all 3 symptoms were significantly higher as compared with the first questionnaire (p< 0.05 for all comparisons).

### 3.3 Timing of medical help-seeking behavior

For almost all symptom categories, approximately half (43.7%-55.5%) of participants sought help approximately 1 month after symptom onset (Table 1). About 80% of participants sought medical help within approximately 1 month for children with any symptom. Only 1.4% to 2.2% of participants waited longer than 1 year to seek help for a child with any symptom.

After the education program, the rate of participants who sought help within 1 week was significantly higher (p=0.001), as compared with the first questionnaire, for all symptom categories except sleeplessness. Those who reported waiting approximately half a year to seek help decreased for all symptom categories (p<0.05).

### 3.4 Factors that predicted a decision not to seek psychiatric medical help

Characteristics that predicted a decision not to seek psychiatric medical help (excepting consultation at a department of internal medicine) were having children with all 3 symptoms, age, and family income (p<0.05) (Table 2). Younger parents were less likely to seek psychiatric medical help. Among parents aged 30 to 39 years, 43.9% did not seek psychiatric medical help. The Bonferroni multiple comparison procedure showed significant differences in the rate between parents aged 30 to 39 years and both those aged 40 to 49 years (p=0.003) and those aged 50 to 59 years (p=0.001). In addition, 51.2% of respondents with a family income less than 11 000 US dollars not seek psychiatric medical help. A lower family income was associated with not seeking psychiatric medical help. The Bonferroni multiple comparison procedure showed significant differences in the rate between parents with a family income of 32 000 to 53 000 US dollars and those with an income greater than 110 000 US dollars (P<0.05).

**Table 2:** Associations between parental characteristic and a decision not to seek medical help for children with sleeplessness, strange behavior, and social withdrawal

	Total	Mental hospital: A			Psychiatric clinic: B			Department of psychosomatic medicine: C			A, B, and C			Department of internal medicine		
	n	n	%	p*	n	%	p*	n	%	p*	n	%	p*	n	%	p*
**Age (years)**				0.528			0.012			0.521			0.006			0.188
30-39	221	187	84.7		179	81.0		119	53.8		97	43.9		203	91.9	
40-49	1904	1564	82.1		1382	72.6		940	49.4		654	34.3		1799	94.5	
50-59	548	461	84.1		381	69.5		278	50.7		170	31.0		525	95.8	
60-69	17	13	76.5		11	64.7		7	41.2		4	23.5		16	94.1	
**Gender**				0.001			0.001			0.001			0.655			0.396
Male	1381	1093	79.1		953	69.0		748	54.2		469	34.0		1311	94.9	
Female	1309	1132	86.5		1000	76.4		596	45.5		456	34.8		1232	94.1	
**Education**				0.715			0.012			0.042			0.072			0.673
Junior high school	25	19	76.0		21	84.0		14	56.0		11	44.0		25	1.0	
High school	766	641	83.7		585	76.4		391	51.0		286	37.3		727	28.6	
Vocational school/junior college	734	610	83.1		540	73.6		333	45.4		241	32.8		696	27.4	
University	1063	875	82.3		736	69.2		559	52.6		361	34.0		1000	39.3	
Graduate school	96	75	78.1		66	68.8		43	44.8		23	24.0		89	3.5	
Other	6	5	83.3		5	83.3		4	66.7		3	50.0		6	0.2	
**Domicile**				0.003			0.196			0.264			0.323			0.980
Hokkaido/Tohoku	304	233	76.6		220	72.4		157	51.6		99	32.6		287	94.4	
Kanto/Sin-Etsu/Hokuriku	1186	980	82.6		848	71.5		593	50.0		407	34.3		1123	94.7	
Tokai/Kinki	822	705	85.8		619	75.3		422	51.3		300	36.5		775	94.3	
Chugoku/Shikoku/Kyusyu/Okinawa	378	307	81.2		266	70.4		172	45.5		119	31.5		358	94.7	
**Marriage status**				0.211			0.415			0.842			0.973			0.799
Unmarried	3	2	66.7		2	66.7		2	66.7		1	33.3		3	100	
Married	2552	2117	83.0		1850	72.5		1278	50.1		879	34.4		2412	94.5	
Bereaved	14	9	64.3		8	57.1		6	42.9		4	28.6		14	100	
Divorced	12	97	80.2		93	76.9		58	47.9		41	33.9		114	94.2	
**Family structure**				0.635			0.837			0.661			0.759			0.982
2 parents	2092	1738	83.1		1523	72.8		1053	50.3		723	34.6		1976	94.5	
1 parent	89	70	78.7		65	73.0		40	44.9		26	29.2		84	94.4	
3 generations	466	383	82.2		332	71.2		232	49.8		162	34.8		442	94.8	
Other	43	34	79.1		33	76.7		19	44.2		14	32.6		41	95.3	
**Employment**				0.001			0.006			0.002			0.283			0.777
Full-time	1373	1091	79.5		960	69.9		726	52.9		458	33.4		1298	94.5	
Part-time	471	409	86.8		360	76.4		205	43.5		152	32.3		449	95.3	
Self-employed	259	211	81.5		186	71.8		137	52.9		99	38.2		241	93.1	
Full-time housewife	542	480	88.6		417	76.9		251	46.3		201	37.1		512	94.5	
Unemployed	45	34	75.6		30	66.7		25	55.6		15	33.3		43	95.6	
**Occupation**				0.535			0.877			0.003			0.154			0.608
Agriculture and forestry	11	10	90.9		8	72.7		7	63.6		5	45.5		11	100	
Production labor service and transportation and communication	772	624	80.8		556	72.0		424	54.9		286	37.0		729	94.4	
Sales and marketing and service industry	160	134	83.8		114	71.3		65	40.6		44	27.5		148	92.5	
Professionals	689	574	83.3		511	74.0		340	49.3		233	33.8		649	94.2	
Other	1058	883	83.5		764	72.2		508	48.0		357	33.7		1006	95.1	
**Family income, (US dollars)**				0.828			0.103			0.020			0.002			0.199
< 11000	41	36	87.8		33	80.5		22	53.7		21	51.2		36	87.8	
11000–32000	196	160	81.6		146	74.5		98	50.0		74	37.8		189	96.4	
32000–53000	502	421	83.9		377	75.1		284	56.6		198	39.4		476	94.8	
53000–110000	1465	1208	82.5		1065	72.7		710	48.5		485	33.1		1379	94.1	
> 110000	486	400	82.3		332	68.3		230	47.3		147	30.2		463	95.3	

* The chi-square test

### 3.5 School help-seeking behavior among parents of junior and senior high school students

Consultation with a homeroom teacher was the most frequent (13.9%-41.4%) school-based help-seeking behavior (Table 1), and consultation with the school nurse was the least frequent (8.5%-12.5%) school-based help-seeking behavior. After the education program, all school-based consultations were significantly more frequent as compared with responses to the first questionnaire (p<0.05 for all comparisons).

## 4. Discussion

Singh *et al*. (2006) reported that demographic factors associated with longer delays in help-seeking were being single, being unemployed, living alone, living in public housing, and ethnic minority status. Another study reported that patients with schizophrenia might not be fully aware that their condition is deteriorating. In addition, they noted that patients living alone tended to be slower to seek a mental health consultation (Koichi *et al.*, 2009). These findings suggest that parents can play an important role in identifying symptoms of schizophrenia in their children, in whom they are well equipped to notice subtle changes. By identifying schizophrenia at an early stage, parents can reduce the time from onset of symptoms to start of treatment, which is important in improving QOL after treatment (Chong *et al.*, 2004). However, parents of junior and senior high school students sometimes might do not seek help when a child has signs of schizophrenia.

Help-seeking among parents has been studied in many countries. One study investigated 34 parents with children aged 2-15 years in London (Sayal *et al.*, 2010), another study enrolled African American mothers (mean age±SD of children: 14±0.8) in rural Georgia (n = 163) (Murry *et al.*, 2011), and a Canadian report studied 506 parents of children aged 4-17 years (Reid *et al.*, 2011). However, our study differed from those earlier investigations because it targeted parents of junior and senior high school students, because the gender and regional distributions of our sample were almost identical to those of the Japanese general population, and because the present study had a reliable, large sample size (n=2690). In addition, to our knowledge, no other study has investigated the effectiveness of a web-based education program that aimed to improve help-seeking behavior among Japanese parents of adolescents. Studies of help-seeking have not yielded consistent results with regard to sex-based, socioeconomic, and ethnic determinants of behavior or the impact of such behavior on treatment delays (Anderson *et al.*, 2010). Help-seeking may differ due to the nature of available medical care, the economy, and/or culture. Our study is therefore important.

We hypothesized that most parents of junior and senior high school students would consult departments of internal medicine. To test this hypothesis, we investigated help-seeking by inquiring about a nonspecific symptom of schizophrenia (sleeplessness), a prodromal symptom (social withdrawal), and a symptom of schizophrenia (strange behavior). We found that the most common (33.1%-50.0%) form of consultation for all symptom categories was at a department of psychosomatic medicine, a field that is concerned with the diagnosis and treatment of medical diseases and their related psychosocial factors, e.g., essential hypertension and arrhythmia, gastric and duodenal ulcer, bronchial asthma, diabetes mellitus, and migraine. Individuals with mental illnesses can be successfully treated in such departments in Japan. These results disagree with those of Jorm *et al*. (2007), who showed that Australian parents (n=2005) of children aged 12-25 years did not universally recognize the potential value of seeking help from mental health professionals.

In the Australian study, parents frequently mentioned general practitioners (GPs) as an intended source of help for their children when asked questions after vignettes portraying either depression, depression with alcohol misuse, social phobia, or psychosis (Jorm *et al.*, 2007). Our results disagree with those findings. In the present study, 21.6% of parents with a child who had a nonspecific symptom of schizophrenia (sleeplessness) and 2.7% of those with a child who had a prodromal symptom (social withdrawal) sought help at a department of internal medicine, which is similar to seeking treatment at a GP. The Japanese medical system permits easy access to specialists. Therefore, patients and their family do not usually have a stable family doctor and can freely seek specialist medical area care.

Our study showed that about 80% of parents of children with symptoms consult a doctor within 1 month of onset. This result differs from a logistic study in Canada (Czuchta *et al.*, 2001), which showed that a mean of 7.33 months elapsed before parents (n=20) sought psychiatric help (including help from either a family doctor, a psychiatrist, or a psychologist). Delays in seeking help can negatively affect the course and treatment of schizophrenia (Waddington *et al.*, 1995; Wyatt, *et al*. 1997; Marshall *et al.*, 2005). Patients may experience such delays in treatment if their parents do not initially consult a medical doctor.

Provision of psychiatric treatment-seeking behavior has been assessed throughout the world (Joa *et al.*, 2008; Tanaka *et al.*, 2003). For example, a Norwegian study showed that an early intervention program reduced DUP in first-episode schizophrenia from 16 to 5 weeks in a health care setting. The program used a combination of easy-access detection teams (DTs) and a massive information campaign (IC) on the signs and symptoms of psychosis (Joa *et al.*, 2008). A previous study in Japan found that an education program significantly improved psychiatric treatment-seeking behavior among workers (p<0.05) (Tanaka *et al.*, 2003). However, the time required in that study was much longer than in our program. In addition, that study did not target parents of junior and senior high school students.

Nicola *et al*. found that accessing information on the internet was associated with increased use of any mental health service, GPs, and mental health professionals (MHPs) (Reavley *et al.*, 2010). Thus, there is evidence that internet-based therapy programs are an effective means of mental health service delivery (Griffiths *et al.*, 2007). Our education program can be viewed over the internet in 13 minutes, which is likely to be more attractive to busy parents in Japan.

## 5. Conclusions

Many parents consulted a physician in a department of psychosomatic medicine when their child experienced symptoms of mental illness. Our web-based education program was useful in promoting medical help-seeking behavior among parents of junior and senior high school students in Japan.
